# The mating pilus of *E. coli* pED208 acts as a conduit for ssDNA during horizontal gene transfer

**DOI:** 10.1128/mbio.02857-23

**Published:** 2023-12-05

**Authors:** Leticia Beltrán, Holly Torsilieri, Jonasz B. Patkowski, Jie E. Yang, James Casanova, Tiago R. D. Costa, Elizabeth R. Wright, Edward H. Egelman

**Affiliations:** 1 Department of Biochemistry and Molecular Genetics, University of Virginia, Charlottesville, Virginia, USA; 2 Department of Molecular Cell Biology, University of Virginia, Charlottesville, Virginia, USA; 3 Department of Life Sciences, Centre for Bacterial Resistance Biology, Imperial College London, London, United Kingdom; 4 Department of Biochemistry, University of Wisconsin-Madison, Madison, Wisconsin, USA; Washington University School of Medicine, Saint Louis, Missouri, USA

**Keywords:** cryo-EM, conjugation pilus, horizontal gene transfer

## Abstract

Bacterial conjugation, a process of horizontal gene transfer, plays a key role in promoting the spread of antimicrobial resistance among human pathogens. The mechanism of conjugation involves the development of a conjugative pilus that forms a physical bridge between two bacterial cells and the subsequent unidirectional transfer of single-stranded DNA (ssDNA) complexed with a protein from the donor to the recipient cell. Atomic structures exist for many of the components of the type IV secretion system (T4SS), responsible for the nucleoprotein secretion, but little is known about the events preceding gene transfer, specifically what is the extent of the participation of the conjugative pilus in ssDNA transfer? There has been a longstanding debate about whether its main role is to bring a donor and a recipient cell into physical juxtaposition and form a mating junction that allows for ssDNA transfer via the T4SS machinery complex or whether ssDNA is actually transferred through the lumen of the pilus. Here, through a combination of maleimide labeling of the conjugative pilus and SeqA-YFP labeling of the transferred ssDNA, we visualize the process of bacterial conjugation in real time. We discover that the conjugative pilus is capable of transferring the ssDNA at a distance, between physically separated cells, and thus conclude that a physical mating junction is not essential for conjugative gene transfer.

**IMPORTANCE:**

Bacteria are constantly exchanging DNA, which constitutes horizontal gene transfer. While some of these occurs by a non-specific process called natural transformation, some occurs by a specific mating between a donor and a recipient cell. In specific conjugation, the mating pilus is extended from the donor cell to make contact with the recipient cell, but whether DNA is actually transferred through this pilus or by another mechanism involving the type IV secretion system complex without the pilus has been an open question. Using *Escherichia coli*, we show that DNA can be transferred through this pilus between a donor and a recipient cell that has not established a tight mating junction, providing a new picture for the role of this pilus.

## INTRODUCTION

Horizontal gene transfer (HGT) is a common process employed by populations of bacteria and archaea to exchange genetic material. For human pathogens, these transfers often involve genes coding for virulence factors and/or antibiotic resistance. The worldwide spread of antimicrobial resistance is a concern for human health as many strains are fast becoming resistant to last-line defense therapeutics. The primary contributor to HGT is bacterial conjugation—a process that involves establishing a physical junction between two bacterial cells, the donor and the recipient, which allows for transfer of DNA. Bacterial conjugation is mediated by the type IV secretion system (T4SS)—a membrane-embedded nanomachine capable of producing a hollow extracellular appendage, known as the conjugative pilus, which projects from the outer membrane of the donor cell and is responsible for the interaction with the recipient cell (1). In Gram-negative bacteria, many high-resolution structures have been determined using cryo-electron microscopy (cryo-EM) for components of the secretion system, including the parts of the T4SS associated with both inner and outer membranes and the pilus, for model organisms such as *Escherichia coli*, *Agrobacterium tumefaciens*, *Klebsiella pneumoniae*, and *Legionella pneumophila* (1–10). The conjugative pilus has a lumen diameter of approximately 15 Å and is composed of many copies of the TraA subunit that folds into an all α-helical structure containing three α-helices. Although the lumen size is an approximation and does not take into consideration the contribution of hydrogens and tightly bound water molecules that might further reduce the diameter, it has been shown that single-stranded DNA (ssDNA) is capable of permeating through pore diameters as small as 10 Å (11). While these structures have led to many new insights regarding the function of these components and ultimately the mechanism of conjugation, many aspects of conjugation are still not very well understood.

The contribution of the F-pilus to bacterial conjugation is still unclear. It has been argued that the F-pilus only serves to bring the donor and recipient cells into direct contact with each other and that the conjugation pilus is fully depolymerized when DNA transfer occurs (12). On the other hand, Babić et al. showed using real-time microscopy that conjugative DNA transfer can occur over distances up to 12 µm (13). While this provided support for the notion that the pilus itself was acting as a conduit for the long-range transfer of the DNA (14), the evidence was indirect. The atomic structures of the F-pilus and related conjugation pili revealed a negatively charged lumen, interpreted as acting to repel negatively charged DNA from the walls of the lumen, potentially lubricating the passage of DNA (4, 5). This was viewed as consistent with the role of the pilus in transferring DNA. However, the structure of the *A. tumefaciens* T-pilus revealed a positively charged lumen (2, 3, 15), seemingly in conflict with this notion of DNA transfer. An explanation was suggested that the properties of the T-pilus lumen may have evolved as a compromise between transferring negatively charged DNA and positively charged effector proteins (3, 16).

A significant question that remains to be answered is how does the DNA get through the recipient cell’s outer membrane, periplasmic space, and inner membrane when cells are conjugating at a distance? There have been studies that suggest that bacterial conjugation is a two-step mechanism for DNA transport whereby the pilus is fully depolymerized and a pilot protein is involved (12). The model called “shoot and pump” implicates the T4SS as a system that shoots the pilot protein, attached to the DNA, through one or both of the recipient cell membranes (12). Given the extreme flexibility of ssDNA, with a persistence length that is only a few bases (17), it is impossible to imagine such a mechanism when mating cells are at a distance from each other, and it is still very difficult to imagine how such a mechanism would be possible even when cells have established a tight mating junction.

Here, using fluorescence light microscopy together with an experimental technique that allows us to see transferred DNA, we show direct evidence that the conjugative pilus is present when mating cells are conjugating at a distance. We show that the length of the pilus is variable when cells have not formed mating pairs. In this study, we use *E. coli* cells harboring the pED208 plasmid that belongs to the IncF family isolated from *Salmonella typhimurium* that constitutively expresses the *tra* genes. With the Alexa-Fluor 568 maleimide labeling of the pED208 pilus TraA subunits, our observations show TraA reinsertion into the inner membrane of the donor cell after depolymerization of the pili, as evidenced by the intense fluorescence of the cell body that is not observed in the mutant strain lacking the pED208 TraA protein. Collectively, our results show that pili, with variable lengths, are present between actively mating pairs.

## RESULTS

### Light microscopy of pED208 WT, pED208 Δ*traA*, and MG1655

**Fig 1:**
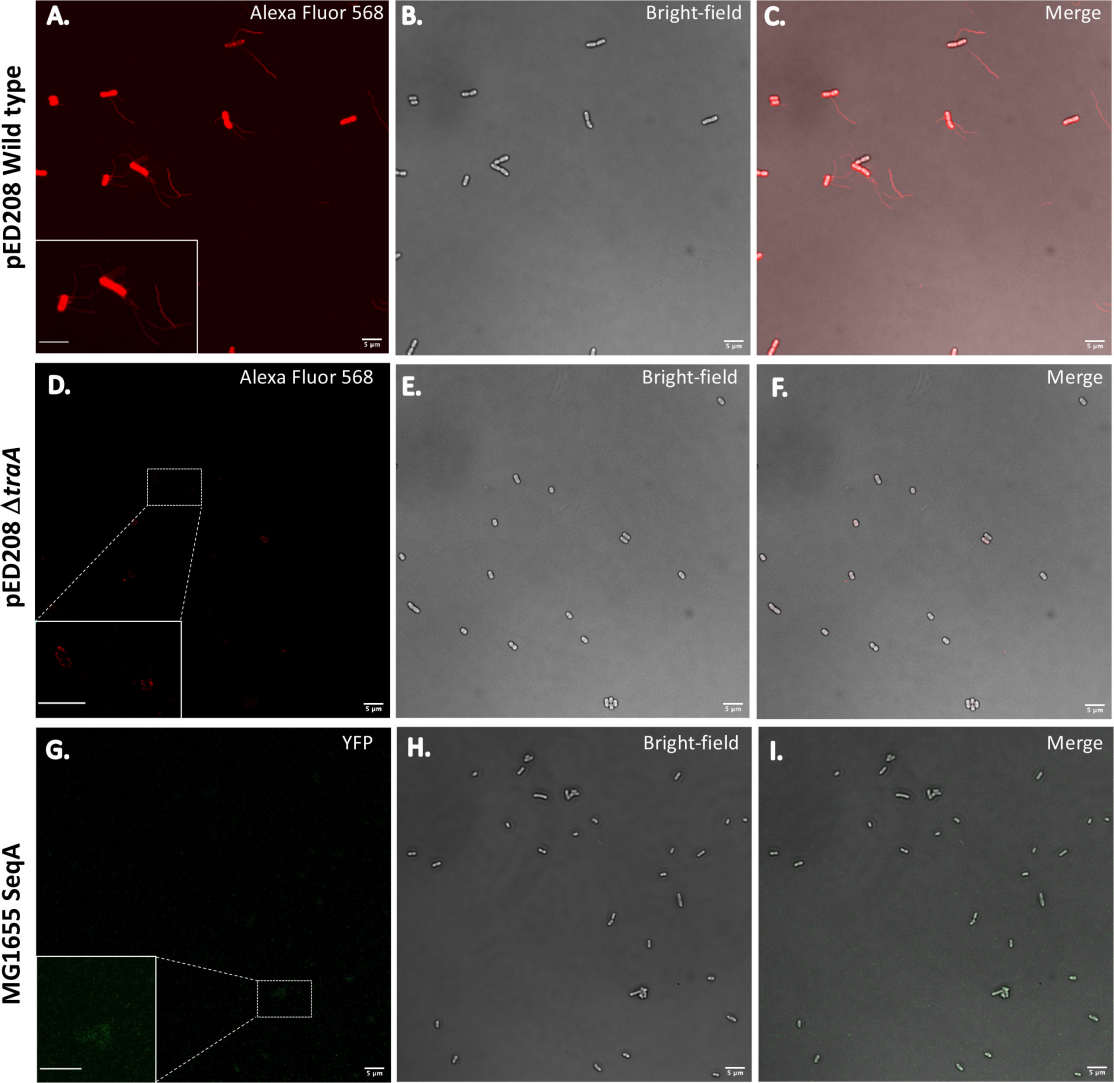
Light microscopy of pED208 WT, pED208 Δ*traA*, and MG1655 SeqA-YFP. (A–C) Fluorescence microscopy image of WT *E. coli* harboring pED208 plasmid (AF-568 labeled cells and F-pili). Brightfield image of the same WT *E. coli* with the pED208 plasmid in (**A**). Overlay of the fluorescence (AF-568 labeled cells and F-pili) and brightfield images (**A, B**) of WT *E. coli* with the pED208 plasmid. (D–F) Fluorescence microscopy image of mutant *E. coli* harboring pED208 Δ*traA* (AF-568 labeled cells and F-pili). Brightfield image of mutant pED208 Δ*traA*. Overlay of the fluorescence (AF-568 labeled cells and F-pili) and brightfield images of mutant pED208 Δ*traA*. (G–I) Fluorescence microscopy image of the recipient cell strain, MG1655 (SeqA-YFP fusion). Brightfield image of the same MG1655 cells. Overlay of the fluorescence and brightfield images of MG1655 (SeqA-YFP fusion). Scale bar is 5 µm.

**Fig 2:**
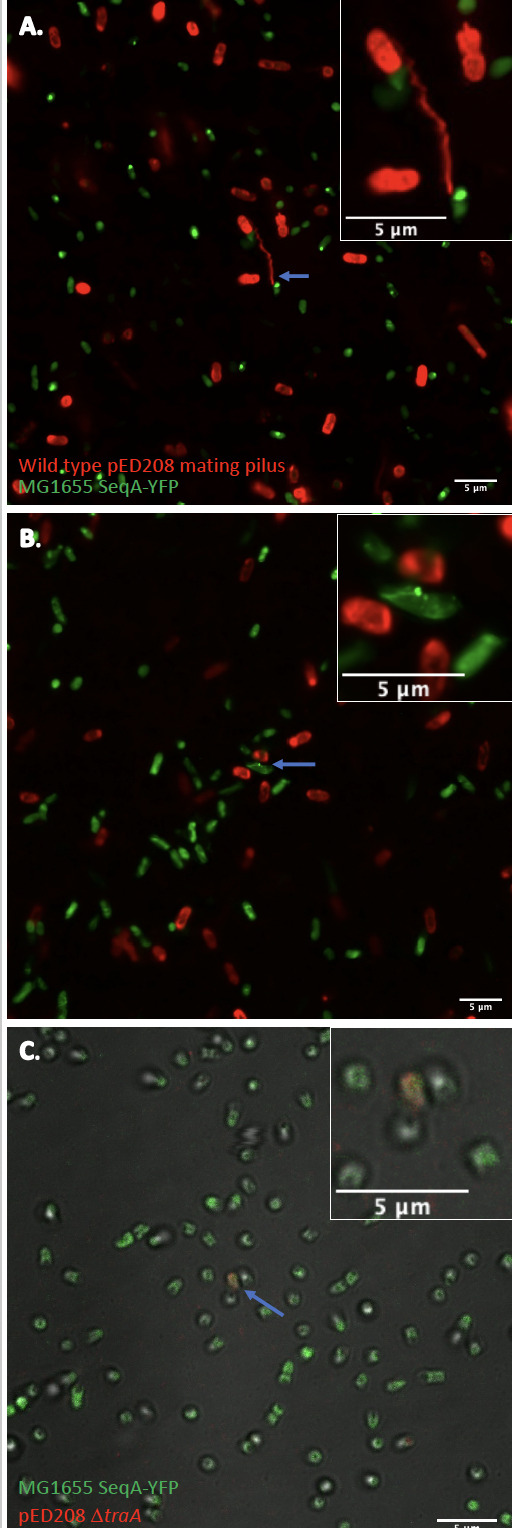
Bacterial conjugation of pED208 WT across multiple spatial scales in comparison with pED208 Δ*traA* . (**A**) The blue arrow points to a WT donor and recipient cell conjugating from a distance (>5 µm). Donor pilus (red) spans the extracellular space and forms an attachment to the recipient cell passing hemimethylated DNA (green puncta). (**B**) Blue arrow points to a WT donor and recipient cell in the process of conjugation after mating pairs have formed. The donor (red) has passed hemimethylated DNA (green puncta) to the recipient cell. Puncta are located at polar ends or the septum. (**C**) Blue arrow points at pair of cells closeby, pED208 Δ*traA* is weakly labeled red as this strain does not express pili, while recipient cells are green and contain no puncta. Scale bar is 5 µm.

Using a labeling scheme that allows us to attach Alexa-Fluor 568 C_5_ maleimide dye to TraA subunits of the donor cell, *E. coli* harboring the pED208 plasmid, we were able to directly target fluorophore attachment to the extracellular donor cell pilus (Fig. 1A through C). Our images (without any quantification) suggest that the number, distribution, and length of pili per donor cell appear to be stochastic, in agreement with other published studies of bacterial conjugal pili (18, 19) (Fig. 1A; Movies S1 to S3; Fig. S5A and B). F-pili are dynamic structures that undergo extension and retraction (19). Extension allows the pilus to survey the surroundings and attach to a recipient cell and then retract, pulling the recipient cell into juxtaposition, followed by the formation of stable mating junctions defined by the contact of the mating pairs’ cell envelopes (Movies S1 to S3; Fig. S5C) (1). All solved structures of conjugation pili, both bacterial and archaeal, show a tight association of lipids with TraA subunits, with a 1:1 stoichiometric ratio for bacterial pili (2–5, 15). It was suggested that one of the functions of this pilin:lipid association would be to facilitate reinsertion of the pilus subunits within the membrane during retraction events (5). However, subsequent studies have shown that there is a 1:1 lipid:pilin association in T-pili, where there has been no evidence of retraction, so the role of the lipid may be more general. Mutant *E. coli* cells lacking the *traA* gene (pED208 Δ*traA*) were labeled with Alexa-Fluor 568 C_5_ maleimide dye that appears to either weakly associate or not all with the cell membranes when compared to the WT pED208 cells that are brightly labeled (20) (Fig. 1A through F; Fig. S1A and B). This suggests a correlation between labeled TraA and reinsertion within the membrane of the donor cells, as discussed in further detail below. It is possible that the weak association of the maleimide dye observed with pilus-deficient pED208 Δ*traA* is due to transiently available cysteines from other proteins found on the membrane surface, since this strain lacks pili compared to the wild-type strain, as shown by negative-stain electron microscopy (Fig. 2A and B). Recipient cells, fully characterized by Babić et al., contain a SeqA-YFP fusion protein that recognizes and binds with high affinity to hemimethylated ssDNA, enabling us to specifically and permanently label only transferred ssDNA (13). It is important to note that dam-positive donor cells generate hemimethylated DNA that is transferred to dam-deficient recipient cells that do not contain or can generate methylated DNA but contain the SeqA-YFP fusion protein that acts as a biosensor for methylated DNA. Diffuse and weak fluorescence throughout the cell body has been attributed to the dam-deficient SeqA-YFP recipient cells (Fig. 1G through I) (13).

### Conjugating bacteria

Similar to observations made by Babić et al., mixing maleimide-labeled donor cells with the SeqA-YFP recipient cell led to the visualization of conjugally transferred DNA in the recipient cells (13). Conjugally transferred DNA is indicated by intense fluorescent foci (green puncta) in recipient cells from SeqA-YFP. The YFP foci seen in recipient cells were often observed at the poles or septum of the cells. During live cell imaging, a mixture of conjugating cells that formed mating junctions, as well as cells conjugating from a distance, was observed (Fig. 2A and B). The donor pilus colocalization with the bright foci of SeqA-YFP within the recipient cell provides direct evidence that the pilus is actually transferring ssDNA between two cells separated by many micrometers. This process of conjugal transfer of DNA through a mating pilus connecting donor and recipient cells has never been directly visualized to the best of our knowledge. When the *traA* gene is knocked out of the pED208 strain, puncta are no longer present in the SeqA-YFP strain when donor and recipient strains are mixed (20) (Fig. 1C; Fig. S1A through C and S2A through C). Interestingly, during the experiments, it was observed that not all donor cells used the same method to transfer ssDNA to the recipient cell. Some mating cells were ~2 µm apart and did not form mating junctions (Movie S1, blue arrow; Fig. S4A and B; Movies S2, S6 and S7; Fig. S5A), while other donor cells used the pilus to probe the volume of the environment attach to a recipient cell and pull it closer (Movie S1, yellow arrow; Movie S4; Fig. S5B). Also observed were mating cells that formed stable mating junctions (Movie S1, green arrow; Movies S3, S6 and S7; Fig. S5C). These observations suggest that not all mating pairs evolve to form mating junctions. Furthermore, donor cells can express multiple F-pili, some of which are seen to attach to recipient cells while others appear to survey the environment (Fig. 1; Movies S1, S4 and S5). Due to the limited resolution of light microscopy, we were unable to directly observe whether any part of the pilus remained once stable mating junctions were formed. A largely depolymerized F-pilus may assist in stabilizing mating pairs together with the TraN-OmpA complex, which is a necessary interaction for efficient conjugation to occur (1, 21, 22). The presence of the F-pilus without T4SS machinery attached has been shown in cryo-EM data (23). Since such pili would presumably be unable to undergo further cycles of polymerization and depolymerization, it was suggested that these static pili might play a role instead in biofilm formation.

### Pilin reinsertion into the cell membrane

Conjugation pili are the only known bacterial filaments where protein subunits are tightly bound to a lipid with a stoichiometric ratio of 1:1. The function of the lipid has been difficult to investigate; however, it has been suggested that the lipid plays a critical role in the stability of the pED208 pilus during both conjugation and biofilm formation (24). When the donor strain carrying the WT pED208 was labeled with maleimide, the pili were easily observed by confocal microscopy, along with strong fluorescence from the cell body (Fig. 1A through C). When the knockout strain lacking the pilin subunit TraA was treated with maleimide, there was a significantly weaker fluorescent signal when compared to that of the WT pED208 strain (Fig. 1A through F; Fig. 2A and B). Since the labeling of the pilin subunits only occurs when the polymerized pilus is extracellular, the presence of labeled subunits in the donor cell body provides direct evidence for the reinsertion of the pilin subunit into the membrane (25).

## DISCUSSION

**Fig 3:**
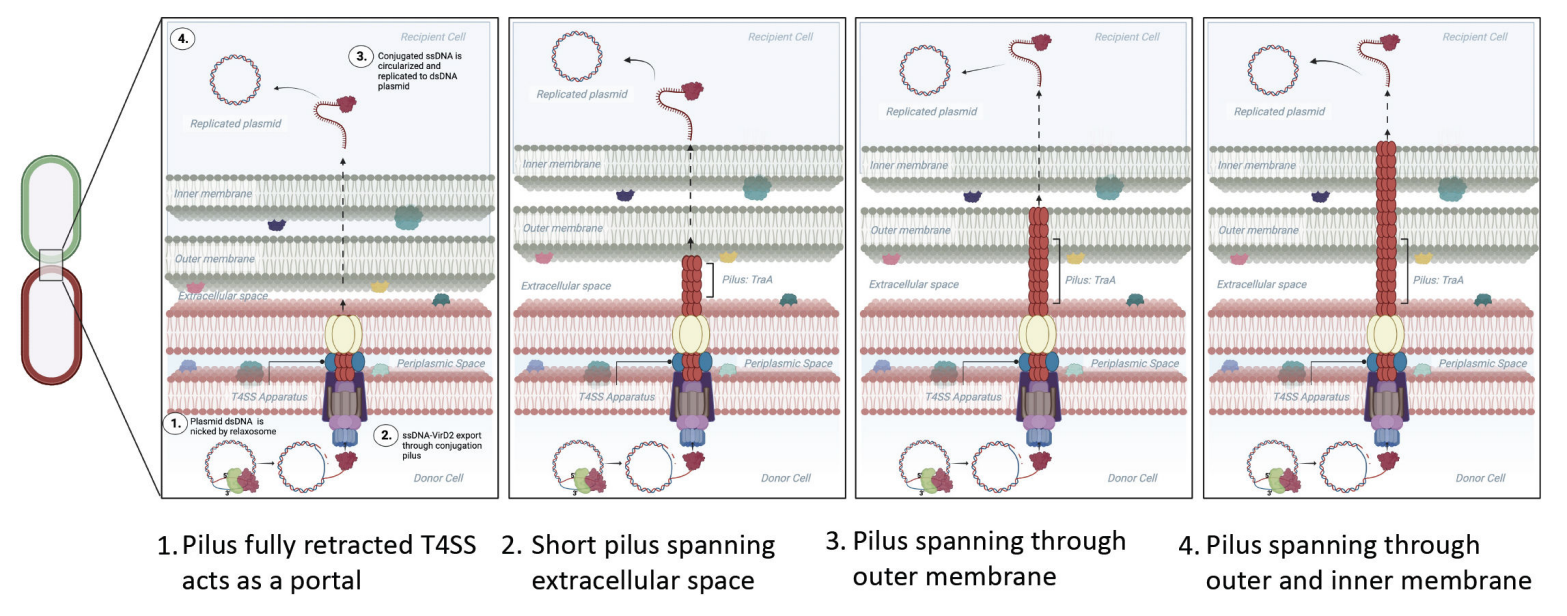
Proposed models of conjugation. Cartoon depicting possible conjugation mechanisms. Models left to right: (1.) model reflects the donor cell T4SS apparatus without the mating pilus. The pilus has been fully depolymerized, and the T4SS acts as the portal for which ssDNA and a pilot protein pass through to the recipient cell cytoplasm. (2.) The mating pilus extends into the extracellular matrix only. The ssDNA traverses, with a pilot protein, through the outer membrane, periplasmic space, and inner membrane to the recipient cytoplasm. (3.) A mating pilus perforates the recipient cell outer membrane, and ssDNA passes with a pilot protein through the periplasmic space and inner membrane to the cytoplasm. (4.) The mating pilus perforates the recipient cell outer membrane, periplasmic space, and inner membrane and delivers ssDNA directly to the cytoplasm.

After ~50 years of investigation, it is evident that there is still much to be understood about bacterial conjugation. Most importantly, we do not understand how the donor cell DNA enters the recipient cell. This study clearly shows that the pED208 pilus is involved in DNA transfer between donor and recipient cells separated by small or large distances. A physiological basis for conjugation at a distance may be supported by the mechanical robustness of the pili as it forms a resilient protective casing (or shaft) for DNA transfer between cells (3, 24). This still leads to many unanswered questions. For example, does the mating pilus penetrate through both the outer and inner membranes of the recipient cell? Or, just the outer membrane? If so, is there a protein or complex that assists in shuttling the DNA into the cytoplasm of the recipient cell? And, when cells have established mating junctions, what happens to the pilus? We describe four possible models for this aspect of bacterial conjugation (Fig. 3). The first shows a fully depolymerized pilus, which could only arise after a stable mating junction has been established. The second, third, and fourth models could exist when mating junctions are established, or they could also apply when mating cells are physically separated and connected only by the pilus. The second model has the pilus running from the donor cell to the outer membrane of the recipient cell, the third model has the pilus extending into the periplasmic space of the recipient cell, while the fourth model has the pED208 pilus penetrating through both the outer and inner membranes of the recipient cell into the cytoplasm.

The first, second, and third models require the presence of a translocation system in the recipient cell for DNA to be pumped into the cytoplasm, as no force provided by the donor cell would be able to propel extremely flexible ssDNA through either the outer or inner membranes of the recipient cell. The role of ComEA/ComEC as a translocation system, moving ssDNA from the periplasm to the cytoplasm, has been directly shown in the process of natural transformation, and it is possible that ComEC functions similarly for the process of bacterial conjugation (26–28). If a recipient cell translocation system is not employed, the fourth model seems the most likely, as the pilus would be positioned to transport ssDNA to the recipient cytoplasm. Such a model for the pilus would be consistent with both transfer at a distance and transfer when stable mating junctions have formed.

Our results provide a direct visualization of pilin reincorporation into the donor cell membrane that must result from depolymerizing labeled pili. While it may be possible that pilin subunits depolymerize at the distal end into the recipient cell membranes, we do not observe any significant fluorescence from the membranes of the recipient cell, making such a possibility unlikely. Our observations show unambiguously that the mating pilus does actually serve as a conduit for DNA transfer, validating the original hypothesis (16). These results provide a rationale for directly targeting the pED208 pilus by therapeutics, and such an approach might aid in combating the spread of antibiotic resistance in bacterial populations.

## MATERIALS AND METHODS

### Generation of the cysteine-rich F-pilus variants

To enhance the fluorescence intensity of the maleimide-labeled F-pilus, the number of surface-exposed thiol groups had to be artificially increased beyond that natively present by the single cysteine in the mature F-pilin polypeptide. Based on the 3D structure of the F-pilus (5), four threonine residues were chosen as candidates for substitution by cysteine residues. Consequently, the T69C, T111C, T112C, and T116C mutants of F-pilin (TraA) were generated through site-directed mutagenesis by inverse PCR according to Takara In-Fusion Snap Assembly protocol. Primer pairs P1 and P2, P3 and P4, P5 and P6, and P7 and P8 were used with pBAD_TraA as template to yield constructs pBAD_TraA:T69C, pBAD_TraA:T111C, pBAD_TraA:T112C, and pBAD_TraA:T116C, respectively. Primer sequences used in the study are listed in Table S1, and all used constructs are summarized in Table S2.

### Complementation assay of cysteine-rich F-pilin variants

To test the generated constructs for functional complementation, we employed a pilus-specific phage spot assay. pBAD_TraA encoding the wild-type F-pilin together with the different substitution variants were transformed into *E. coli* strain DH5α harboring the pilin-deficient pED208:Δ*traA* plasmid and grown to OD_600_ = 0.5. Expression was induced with 0.2% (wt/vol) L-arabinose and allowed to proceed for 2 hours at 37°C before being plated on LB-agar plates supplemented with 0.2% (wt/vol) L-arabinose. Then, 2 µL of the F-pilus-specific phage F1 was spotted on top of each of the freshly dispersed bacterial inocula, and the plates were incubated at 37°C overnight. The next day, formation of bacteriophage plaques on the plates was examined. Positive complementation with the F-pilin and substitutions, e.g., presence of the F-pilus, was determined when F1 phage plaques were observed. Cells harboring the pBAD_TraA:T116C variant produced plaques identical to those with the wild-type pBAD_TraA; therefore, this construct was chosen for further maleimide labeling and imaging.

### Cultivation of *E. coli* strain harboring pED208 plasmid and labeling of pilus, pED208 Δ*traA,* or dam-deficient seqA-YFP strain

A culture of *E. coli* DH5α(F^+^) dam^+^ harboring the pED208 plasmid was grown at 37°C and 180 RPM to an OD_600_ of ~1.1 in 100 mL of LB media containing 50 µg/mL kanamycin and 100 µL of 0.1% ampicillin. A total of 2 mL of 10% arabinose was added for a final concentration of 0.2% to induce the pBAD plasmid expressing the TraA cysteine knock-in.

A culture of *E. coli* JE2571(F^+^) harboring the pED208 plasmid was grown at 37°C and 180 RPM to an OD_600_ of ~1.1 in 100 mL of liquid LB with 50 µg/mL kanamycin.

A culture of *E. coli* MG1655 (F^-^) dam^-^ harboring the SeqA-YFP fusion gene was grown at 37°C and 180 RPM to an OD_600_ of ~0.6 in 100 mL of liquid LB with 25 µg/mL chloramphenicol.

### Negative-stain transmission electron microscopy

A total of 2 µL of sample containing either of *E. coli* DH5α(F^+^) harboring the pED208 + pBAD inducer, pED208 Δ*traA*, or *E. coli* dam-deficient seqA-YFP strain was applied to a 200-mesh carbon-coated grid and stained with 2% uranyl acetate and examined by transmission electron microscopy using a Tecnai-T12 at 80 kV.

### Fluorescence microscopy and image analysis

A total of 5 µL of sample containing either Alexa-Fluor 568 labeled *E. coli* DH5α(F^+^) + pBAD donor cells mixed with the recipient *E. coli* dam-deficient seqA-YFP strain or pED208 Δ*traA* donor cells mixed with recipient *E. coli* dam-deficient seqA-YFP strain or samples only containing *E. coli* DH5α(F^+^) harboring the pED208 + pBAD, pED208 Δ*traA*, or *E. coli* dam-deficient seqA-YFP strain was applied to 25 × 75 mm 1.0-mm-thick glass microslide with or without an agar pad and covered with a 22 × 22 mm micro cover glass slide. The specimens were imaged using a Nikon Ti2-E inverted microscope with AX-R confocal microscopy system, 25 mm FOV resonant scanner, 25 mm FOV Galvano scanner, and an NSPARC detector unit. Images were captured using photomultipliers and the Nikon NIS Elements C software. Live cell imaging was performed at 23°C, and the samples were viewed using a 100×/NA1.52 oil objective with perfect focus during live image acquisition. Captured images and movies were processed using Elements Denoise.ai followed by compiling in FIJI software (29).

## AFTER EPUB

[This article was published on 5 December 2023 without the supplemental material. The supplemental material was added in the current version, posted on 4 January 2024.]

## SUPPLEMENTAL MATERIAL

Movie S1 mbio.02857-23-s0001.avi The blue arrow denotes the location for conjugation at a distance.

Movie S2 mbio.02857-23-s0002.avi Zoomed-in field of view of the region of interest, blue arrow from Movie S1, which shows conjugation happening at a distance.

Movie S3 mbio.02857-23-s0003.avi A zoomed-in field of view of the region of interest, green arrow from Movie S1, showing conjugation between two cells which appear to have formed stable mating pairs.

Movie S4 mbio.02857-23-s0004.avi A zoomed-in field of view of the region of interest, yellow arrow from Movie S1.

Movie S5 mbio.02857-23-s0005.avi The yellow arrow marks the point where a red punctum is colocalized on a recipient cell.

Movie S6 mbio.02857-23-s0006.avi Conjugation at a distance in a large field of view.

Movie S7 mbio.02857-23-s0007.avi The zoomed-in field of view, black box from Movie S6.

Supplementary Material mbio.02857-23-s0008.pdf Supplemental tables, figures, and movie legends.

ASM does not own the copyrights to Supplemental Material that may be linked to, or accessed through, an article. The authors have granted ASM a non-exclusive, world-wide license to publish the Supplemental Material files. Please contact the corresponding author directly for reuse.
