# Supplementary material for: The mating pilus of E. coli pED208 acts as a conduit for ssDNA during horizontal gene transfer
Source: mBio. 2023 Dec 5;15(1):e02857-23. doi: 10.1128/mbio.02857-23 (PMC10790687; doi:10.1128/mbio.02857-23)
Supplement: Supplementary Material — Supplemental tables, figures, and movie legends. [file mbio.02857-23-s0008.pdf]

Table S1. List of primers used in the study

Primer	In-house label	DNA sequence 5' to 3'
P1	T69C F	GAAAGCCTGCTTCGGTGCCGACTCATTCG
P2	T69C R	CCGAAGCAGGCTTTCACATCATCCTTGCCC
P3	T116C F	CGGTCTTTGCTTCATCAAATGATAAGAGAGCTTGG
P4	T116C R	ATGAAGCAAAGACCGACGGTAGTGAAGAC
P5	T112C F	CTTCACTTGCGTCGGTCTTACCTTCATCAAATG
P6	T112C R	CCGACGCAAGTGAAGACGATAACCACAACCAG
P7	T111C F	CGTCTTCTGCACCGTCGGTCTTACCTTCATC
P8	T111C R	ACGGTGCAGAAGACGATAACCACAACCAGGC

Table S2. List of constructs used in this study

Name of construct	Description	Source
pBAD_TraA	pBADM-11 cloned with *traA* gene from pED208 plasmid under arabinose-inducible promoter, Amp^R	Costa et al., 2016
pBAD_TraA:T69C	T69C substitution mutation of pBAD_TraA	This study
pBAD_TraA:T111C	T111C substitution mutation of pBAD_TraA	This study
pBAD_TraA:T112C	T112C substitution mutation of pBAD_TraA	This study
pBAD_TraA:T116C	T116C substitution mutation of pBAD_TraA	This study

Supplemental Movie 1. The blue arrow denotes the location for conjugation at a distance. A pilus is seen to attach to the recipient cell and transfer hemimethylated ssDNA recognized by seqA-YFP producing puncta (top). The yellow arrow indicates a donor cell with the appearance of two separate pili, one pilus attaches, then retracts, pulling in a recipient cell (middle). The green arrow shows conjugation between mating pairs (bottom). The scale bar is 5 μm.

Supplemental Movie 2. A zoomed in field of view of the region of interest, blue arrow from Supplemental Movie 1, which shows conjugation happening at a distance. The donor cell pilus is seen to attach to the recipient cell and transfer hemimethylated ssDNA recognized by seqA-YFP producing puncta.

Supplemental Movie 3. A zoomed in field of view of the region of interest, green arrow from Supplemental Movie 1, showing conjugation between two cells which appear to have formed stable mating pairs.

Supplemental Movie 4. A zoomed in field of view of the region of interest, yellow arrow from Supplemental Movie 1, which shows the appearance of two separate pili, one pilus attaches, then retracts, pulling in a recipient cell, followed by green fluorescence colocalized to the event.

Supplemental Movie 5. The yellow arrow marks the point where a red punctum is colocalized on a recipient cell. Frame 0:03.79 reveals a visible donor cell pulling itself toward the recipient cell. Top left yellow arrow marks a donor cell which has two separate pili which appear to be surveying the environment at frame 0:04.88. The scale bar (far right) is 5 μm and the elapsed time is 1 minute every 10 seconds.

Supplemental Movie 6. This movie shows conjugation at a distance in a large field of view. The black box indicates the region of interest where conjugation events, both mating at a distance and nearby, are occurring (Supplemental Movie 7). Snapshots of these two conjugation events were taken from frames 10 and 21 which include the time in seconds the event occurs in the movie. In frame 10, the donor cell pilus is labeled by C5 maleimide dye Alexa-Fluor 568 and the cell body can be observed to fluoresce red. In frame 21, the cell body of the donor cell is not labeled, only the long pilus is, and the recipient cell body has green background fluorescence with ssDNA indicated by a green punctum.

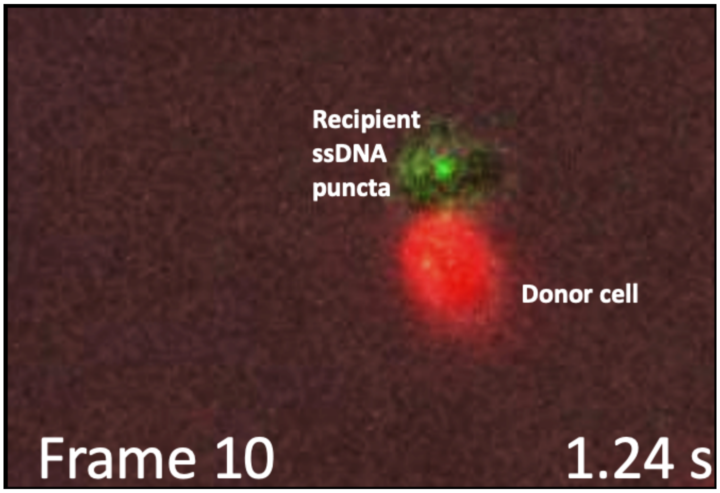

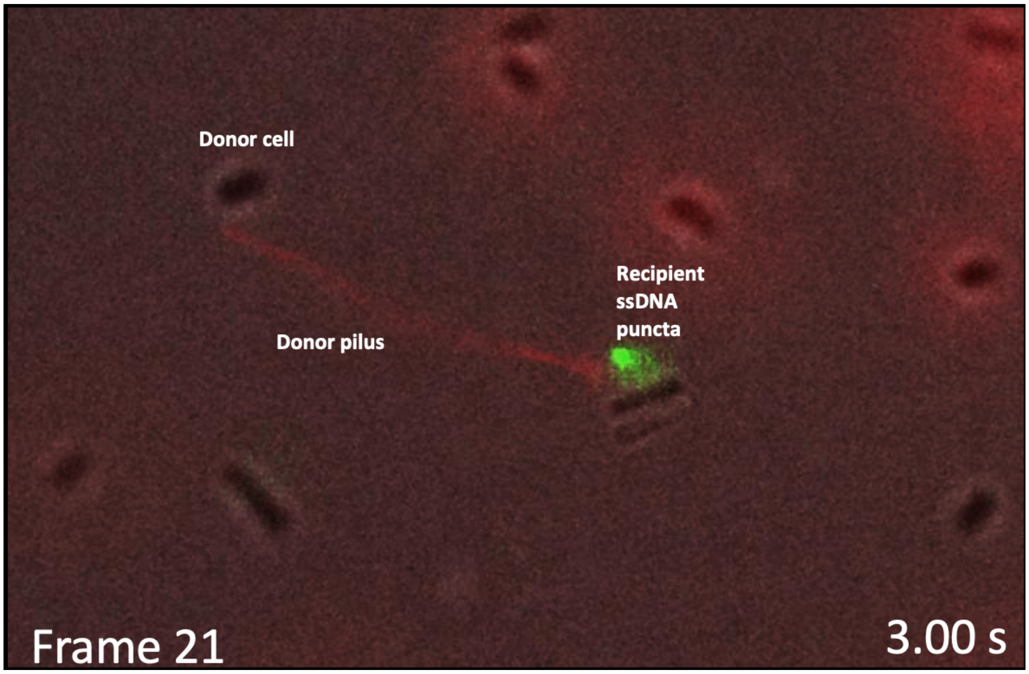

Supplemental Movie 7. The zoomed-in field of view, black box from Supplemental Movie 6.

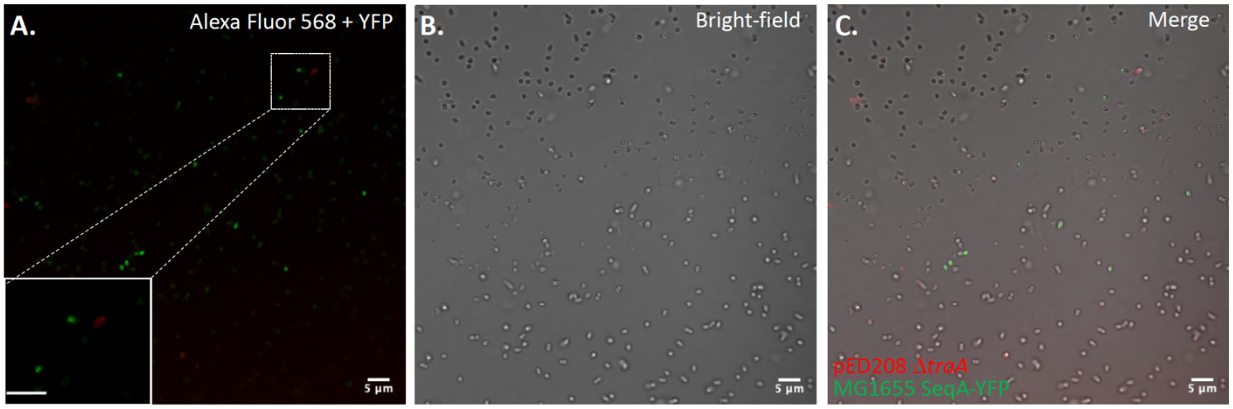

Supplemental Figure 1. pED208 Δ*traA* strain mixed with MG1655 SeqA-YFP recipient strain. (A) Fluorescence microscopy image of *E. coli* harboring pED208 Δ*traA* plasmid (labeled with AF 568) mixed with MG1655 SeqA-YFP recipient strain. **(B)** Brightfield image of pED208 Δ*traA* plasmid mixed with MG1655 SeqA-YFP recipient strain. **(C)** Merge of the fluorescence and brightfield images of *E. coli* harboring pED208 Δ*traA* mixed with MG1655 SeqA-YFP recipient strain.

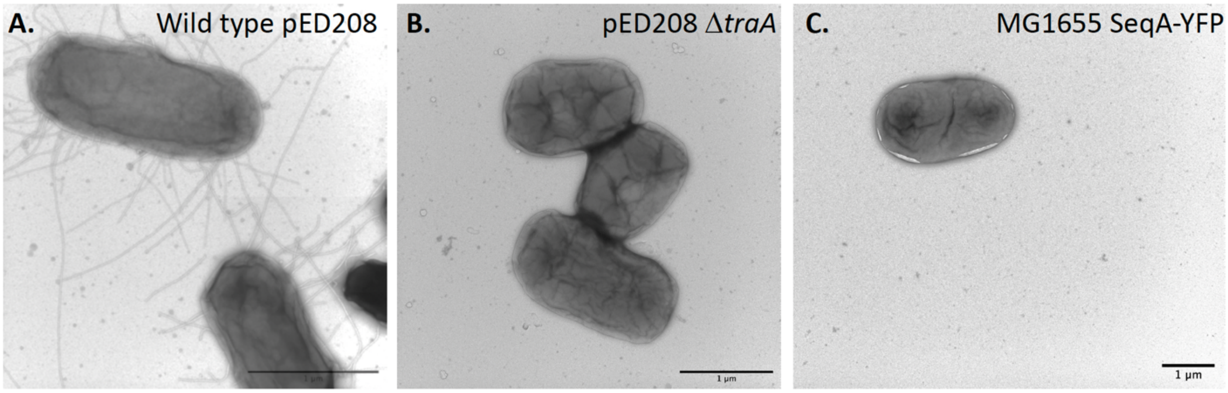

Supplemental Figure 2. **Negative stain TEM images. (A)** Wild-type *E. coli* strain JE2571 harboring pED208 plasmid and labeled with Alexa Fluor 568. **(B)** Mutant *E. coli* strain JE2571 Δ*traA*, pED208 Δ*traA* and labeled with Alexa Fluor 568. **(C)** *E.coli* strain MG1655 nalR dam::FRT seqA-yfp::Cm.

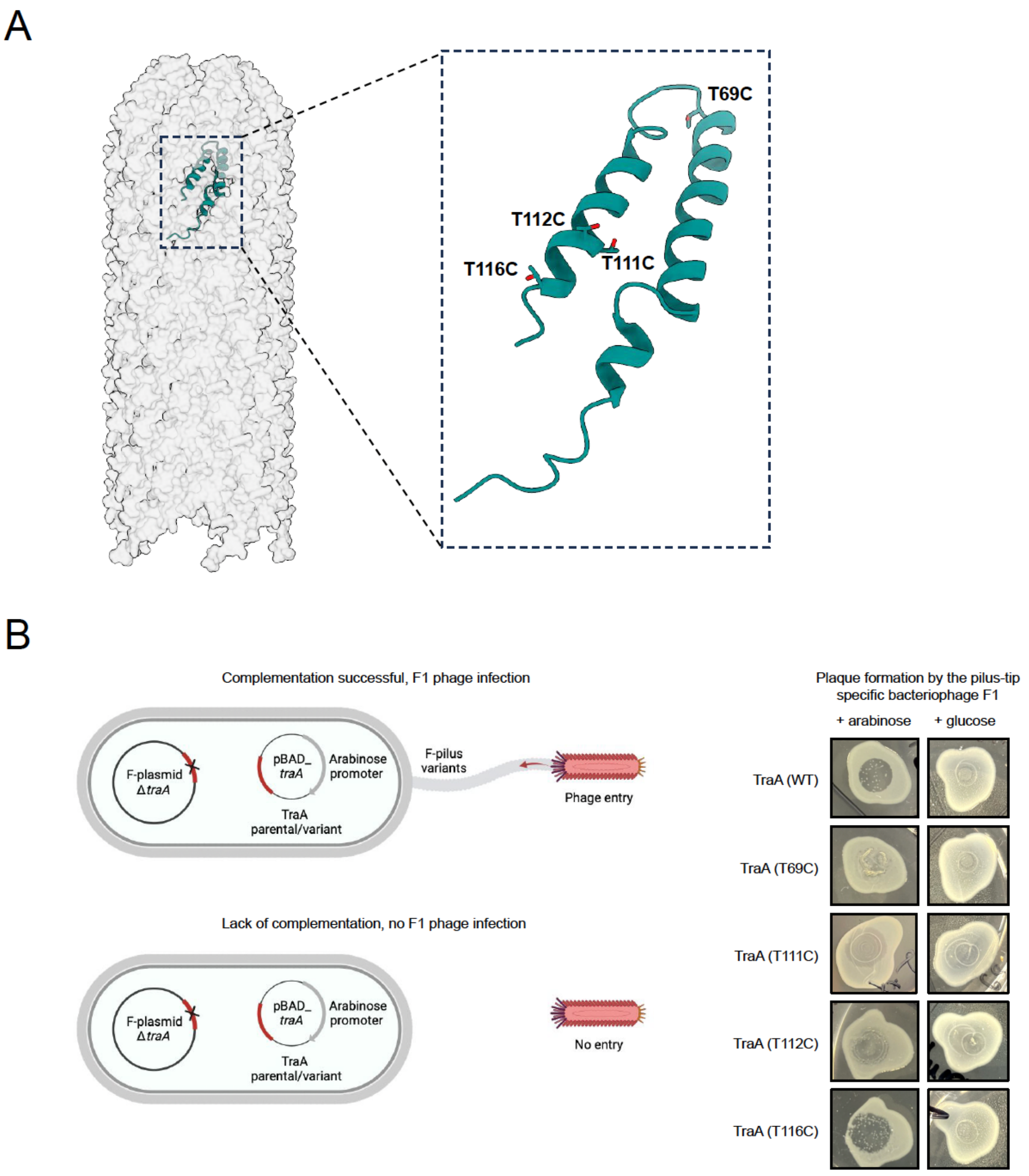

Supplemental Figure 3. Schematic for labeling pED208 TraA subunits. (A) PDB: 5LEG pilus of wild-type *E. coli* strain JE2571 harboring pED208 plasmid. The yellow stars indicate the maleimide derivative targets of Alexa Fluor 568, residues T116 & C79 for attachment. The predictive relative surface accessibility (RSA) is 67% and 54% for T116 and C79, respectively. **(B)** F1 phage plaque assay to validate that the point mutation cysteine knock-in for T116 was successful. F1 phage binds to accessible F-pili. Clear F1 phage plaques indicate successful knock-in.

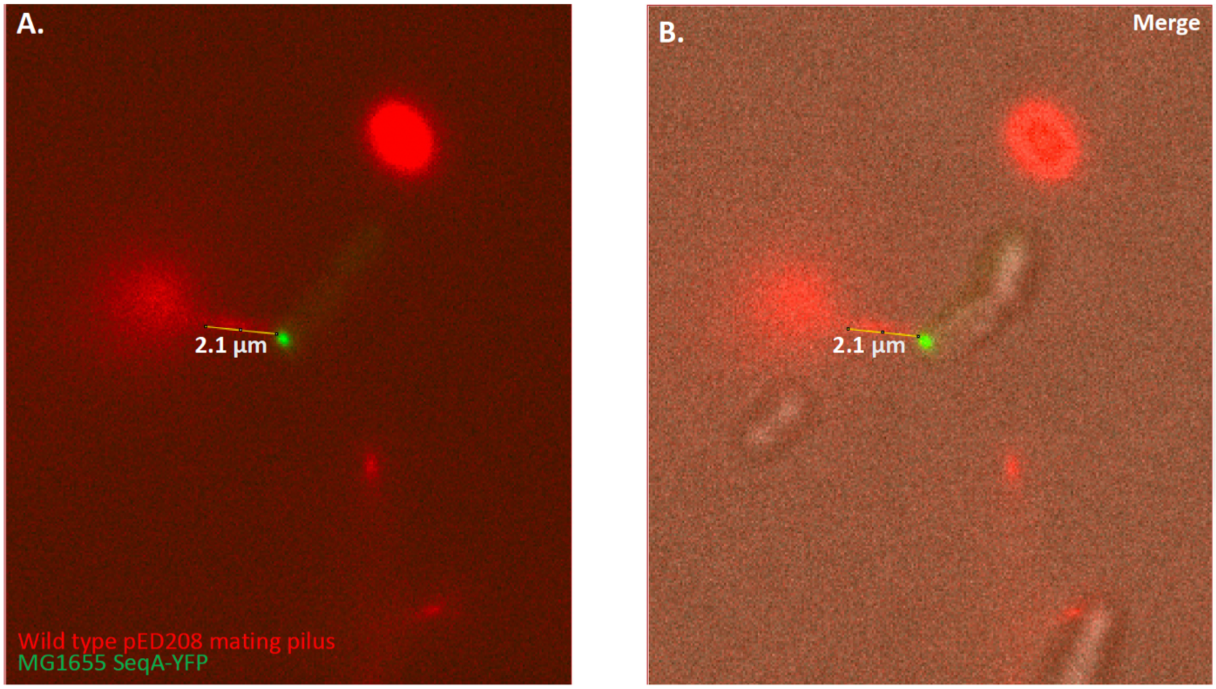

Supplemental Figure 4. Measurement of mating pilus when mating pairs have not formed mating junctions but transferred ssDNA at a shorter distance ~2 micrometers. **(A)** Fluorescence images of donor cell pED208 (AF 568 – red) and recipient cell (SeqA-YFP fusion - green). Bright puncta indicates the transfer of ssDNA to recipient cell. **(B)** Brightfield overlay of panel A for better visualization of cells.

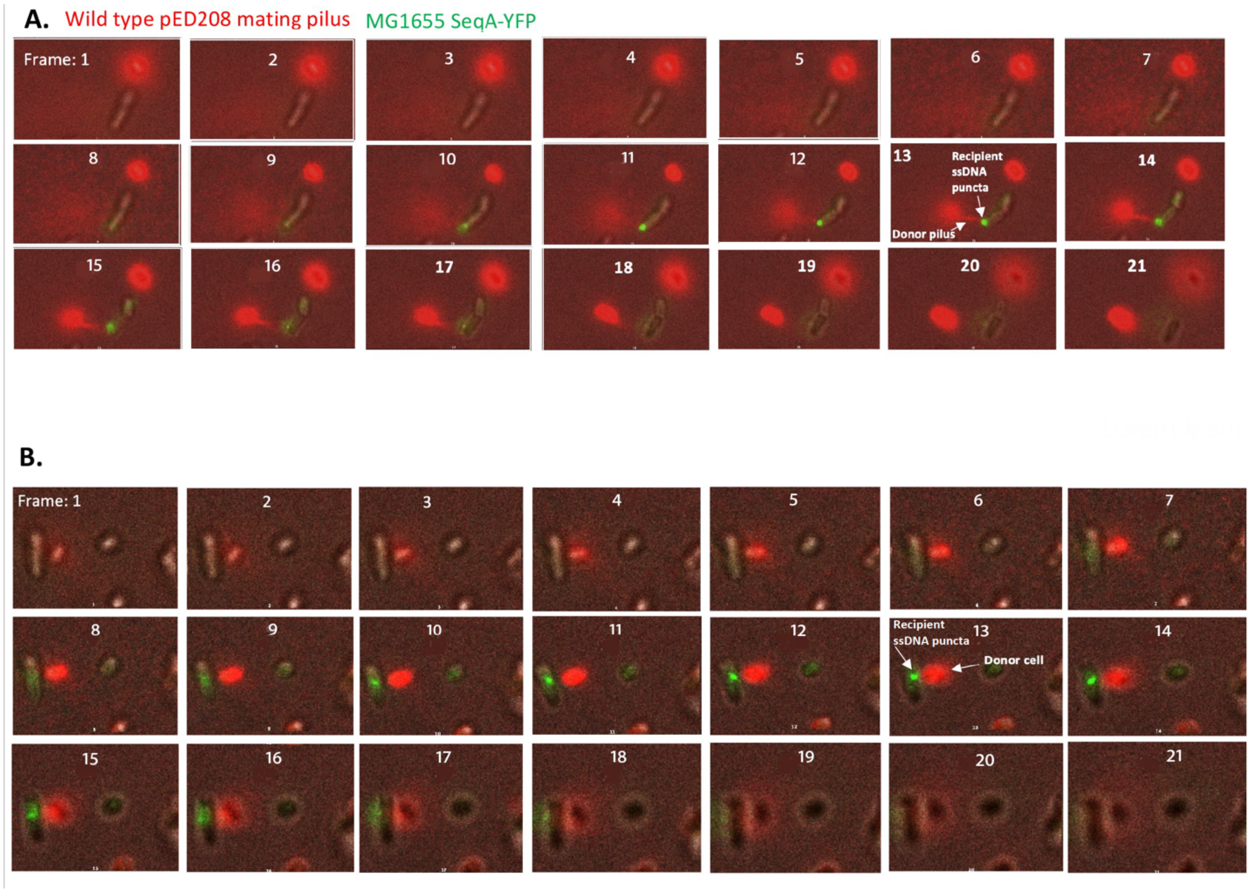

Supplemental Figure 5. Frame-by-frame montage of the three different events happening in Movie 1 at blue, yellow, and green arrows respectively. **(A)** At the blue arrow, conjugation happening at a distance between donor (red) and recipient (green) cells. Frames 10-17 show green puncta, the SeqA-YFP which specifically recognizes and targets hemi-methylated ssDNA. By frames 12-17 the labeled donor cell pilus can be seen to colocalize in the same region as the green puncta of the recipient cell. After frame 17 the green puncta slowly disappears which is likely the bacterial cell leaving the z-plane of view. **(B)** At the yellow arrow, donor cell pili are seen to attach to recipient cell and pull the recipient cell towards itself. The donor cell body is out of the z-plane and is not directly observed to be connected to the pili, however, it is evident that the recipient cell is being pulled out of the plane of view in frames 7-21. **(C)** At the green arrow, conjugation occurring once mating junctions have formed. Donor cell appears to form an attachment to recipient cell starting in frame 1 and transfers the ssDNA to the recipient cell in frame 10, indicated by formation of green puncta. The fluorescence appears to wane after frame 16 and is likely due to cells leaving the z-plane of view.
